# Supplementary material for: The Effect of GD1a Ganglioside-Expressing Bacterial Strains on Murine Norovirus Infectivity
Source: Molecules. 2020 Sep 7;25(18):4084. doi: 10.3390/molecules25184084 (PMC7571017; doi:10.3390/molecules25184084)
Supplement: Supplementary file 1 [file molecules-25-04084-s001.pdf]

molecules

Supplementary materials: The effect of GD1a ganglioside-expressing bacterial strains on murine norovirus infectivity

Yifan Zhu [1], Hiroki Kawai [2], Satoshi Hashiba [2], Mohan Amarasiri [3,4], Masaaki Kitajima [2], Satoshi Okabe [2], and Daisuke Sano [1,3]*

[1] Department of Frontier Sciences for Advanced Environment, Graduate School of Environmental Studies, Tohoku University, Aoba 6-6-06, Aramaki, Aoba-ku, Sendai, Miyagi 980-8579, Japan; zhu.yifan.t1@dc.tohoku.ac.jp

[2] Division of Environmental Engineering, Faculty of Engineering, Hokkaido University, North 13 West 8, Kita-ku, Sapporo, Hokkaido 060-8628, Japan; mkitajima@eng.hokudai.ac.jp, sokabe@eng.hokudai.ac.jp

[3] Department of Civil and Environmental Engineering, Graduate School of Engineering, Tohoku University, Aoba 6-6-06, Aramaki, Aoba-ku, Sendai, Miyagi 980-8579, Japan; daisuke.sano.e1@tohoku.ac.jp

[4] Department of Health Science, School of Allied Health Sciences, Kitasato University, 1-15-1 Kitasato, Sagamihara-Minami, Kanagawa 252-0373, Japan; mohan@kitasato-u.ac.jp

* Correspondence: daisuke.sano.e1@tohoku.ac.jp; Tel.: +81-022-795-7481

# Experimental protocols for identifying and isolating GD1a-positive bacterial strains from the sewage sample.

Flow cytometry

The sewage sample was taken at a wastewater treatment plant on Sousei river, Hokkaido, Japan. The sample was first filtered by a 20 μm membrane (25AS020AS; Advantec, Japan) to remove large solids, the permeate was then centrifuged at 6000×g for 5 min. After discarding the supernatant, the pellet was resuspended with DPBS. The cell concentration was determined by measuring the OD_{600} value. Based on the result, roughly 10^7 cells were transferred to a 1.5 mL centrifugation tube and centrifuged again at 6000×g for 5 min, the pellet was resuspended and well pipetted with 15% BSA/PBS solution. The suspension was stirred at 800 rpm for 45 min at room temperature for blocking followed by centrifugation (6000×g, 5 min). After discarding the supernatant, the pellet was then resuspended with the anti-GD1a antibody (MAB5606; EMD Millipore) diluted 1:100 by antibody dilution buffer (5% BSA/PBS + 0.05% Tween20). After sufficient pipetting, the suspension was stirred at 800 rpm for 25 min at room temperature for mixing followed by centrifugation (6000×g, 5 min). The pellet was washed twice with antibody dilution buffer resuspension and centrifugation (6000×g, 5 min). Then, the pellet was resuspended with the fluorescence-labeled rabbit anti-mouse IgG (H+L) (28164-05-H555; AnaSpec, USA) diluted 1:100 by antibody dilution buffer. After sufficient pipetting, the suspension was stirred at 800 rpm for 25 min at room temperature and shading condition followed by centrifugation (6000×g, 5 min). The pellet was washed twice with antibody dilution buffer, and the final volume of the suspension was adjusted to 500 μL by DPBS. The sample was then loaded into a desktop fluorescent activated cell sorter for analysis (JSAN; Bay Bioscience, Japan). The control group was prepared following the same procedure only without the step of resuspension with diluted anti-GD1a antibody. The result of the sewage sample was compared with that of the control group, the bacterial strains that showed the fluorescent intensity higher than background level in the presence of anti-GD1a antibody were considered as possible GD1a-positive strains. R2A medium was added to the wells that contained candidate strains, the culturable strains were purified by the streak plate method and cultured at 37°C for the following experiments.

Chemiluminescent dot blot assay

Tris buffer saline (TBS), TBS-Tween (TBST), 1% blocking buffer, and 0.5% blocking buffer were prepared according to the formulas shown in Table S1. PVDF membrane was submerged in methanol for 1 min, and 3 μL bacteria suspension was dripped on the membrane for immobilization. The membrane was then washed by TBS for 5 min followed by submerged in 1% blocking buffer in a shaker. The membrane was washed by TBST for 3 times, 5 min each, then submerged in anti-GD1a antibody diluted 1:1000 by 0.5% blocking buffer with gentle shaking for binding reaction. After washed with TBST and 0.5% blocking buffer for 10 min each, the membrane was submerged in goat anti-mouse HRP-conjugated antibody (A90-101P; Bethyl Laboratories, USA) diluted 1:1000 by 0.5%

blocking buffer. After the reaction, the membrane was washed by TBST four times, 15 min each. Norovirusex® ECL (WP20005; Thermo Fisher, USA) was added on the membrane following the manufacturer's instruction, after sitting for 1 min, the membrane was analyzed by ImageQuant LAS 4000 (GE Healthcare, USA) for the chemiluminescent signal. The control group was prepared by extracting the cellular lipids from RAW 264.7 cells using a previously described method [52,79] with minor adjustments. Briefly, confluent RAW 264.7 cells washed twice by DPBS followed by centrifugation (2000 rpm, 5 min), the pellet was then resuspended with 200 μL methanol and 40 μL MilliQ water, another 100 μL methanol and 150 μL chloroform were added to the suspension, the mixture was sonicated for 15 min followed by centrifugation (4000 g, 30 min). After removing the chloroform layer and water layer, the pellet was resuspended with 100 μL 0.9% NaCl solution and centrifuged (4000 g, 30 min), the supernatant was used as the extracted cellular lipids.

Table S1 Formula of the buffers used in dot blot assay

Tris-buffered saline (TBS), pH 7.5	
50 mM $C_4H_{11}NO_3$	6.05g/L
150 mM NaCl	8.76g/L
TBS-Tween (TBST)	
TBS	1L
Tween 20	1mL
1% blocking buffer	
TBS	90mL
10x Western blocking reagent	1mL
0.5% blocking buffer	
TBS	50mL
1% blocking buffer	50mL

Western blotting

The cell culture suspension of the candidate bacteria strains selected by dot plot assay was centrifuged at 6000 × g for 5 min at 4°C. The supernatant was discarded and the pellet was resuspended with 5 mL 0.9% NaCl and vortexed for 2 min. The suspension was cultured for 20 min at 37°C under 150 rpm stirring and then centrifuged for 10 min at 8000 × g, the pellet was resuspended again with 5 mL 0.9% NaCl and vortexed for 3 min. The suspension was transferred to 90°C water bath for 45 min and then the centrifuged at 12000×g for 20 min at 4°C. The supernatant was recovered and freeze-dried as tightly bound extracellular polymeric substances (TB-EPS), followed by resuspension with MilliQ water. Western blot was carried out using the NuPAGE® electrophoresis

system (Thermo Fisher, USA) following the manufacturer's instruction, the anti-GD1a antibody and the goat anti-mouse HRP-conjugated antibody were used as the primary antibody and the secondary antibody, respectively. The PVDF membrane was analyzed by ImageQuant LAS 4000 for the chemiluminescent signal.

16S rRNA gene sequencing

The colony of each strain formed on R2A agar medium was dissolved with 100 μL 0.05M NaOH solution and heated by a thermal cycler at 95 ℃ for 15 min. The mixture was then centrifuged at 1000 rpm for 5 min, 10 μL supernatant was recovered and diluted 1:100 with MilliQ water. The sample was then amplified by PCR using primers 27f and 1492r and Ex Taq® PCR kit (RR001A; Takara Bio, USA). The PCR mixture formula, the sequence of primers, and the PCR condition were shown in Table S2 and S3. The PCR product was purified using FastGene Gel/PCR extraction kit (FG-91302; NIPPON Genetics, Japan) following the manufacturer's instruction, the final product was adjusted to 30-120 ng. After mixing with primers 27f, 530f, 907r, and 1492r, the samples were sent to Eurofins Japan for 16S rRNA gene sequencing.

Table S2 Formula of PCR mixture

Constituent	Volume
10X Extaq buffer	4 μL
dNTP	3.2 μL
F primer	0.2 μL
R primer	0.2 μL
Extaq	0.2 μL
Sample	2 μL
MilliQ water	30.2 μL
Total volume	40 μL

Table S3 Sequences of primers and PCR condition

Name	Sequence
27f	AGAGTTTGATCCTGGCTCAG
530f	GTGCCAGCMGCCGCGG
907r	CCGTCAATTCMTTTRAGTTT
1492r	TACGGYTACCTTGTTACGACTT

Cycle	Temperature	Duration
1 cycle	95 ℃	2 min
30 cycles	95 ℃	1 min
	55 ℃	1 min
	72 ℃	1 min
1 cycle	72 ℃	5 min
	4 ℃	∞
